# Identification of Antagonistic Action of Pyrrolizidine Alkaloids in Muscarinic Acetylcholine Receptor M1 by Computational Target Prediction Analysis

**DOI:** 10.3390/ph17010080

**Published:** 2024-01-08

**Authors:** Sara Abdalfattah, Caroline Knorz, Akhtar Ayoobi, Ejlal A. Omer, Matteo Rosellini, Max Riedl, Christian Meesters, Thomas Efferth

**Affiliations:** 1 Department of Pharmaceutical Biology, Institute of Pharmaceutical and Biomedical Sciences, Johannes Gutenberg University, Staudinger Weg 5, 55128 Mainz, Germany; saabdelf@uni-mainz.de (S.A.); c.knorz@outlook.com (C.K.); ayoobial@uni-mainz.de (A.A.); ejlaomer@uni-mainz.de (E.A.O.); mroselli@uni-mainz.de (M.R.); 2 Department of Plant Sciences, Faculty of Biological Sciences, Alzahra University, Tehran 19938 93973, Iran; 3 Institute for Medical Informatics, Statistics and Epidemiology, University of Leipzig, 04107 Leipzig, Germany; maxriedl@outlook.com; 4 High Performance Computing Group, University of Mainz, 55131 Mainz, Germany; meesters@uni-mainz.de

**Keywords:** alkaloids, computational biology, herbal medicine, natural products, neurotoxicity, phytotherapy, virtual drug screening

## Abstract

Pyrrolizidine alkaloids (PAs) are one of the largest distributed classes of toxins in nature. They have a wide range of toxicity, such as hepatotoxicity, pulmonary toxicity, neuronal toxicity, and carcinogenesis. Yet, biological targets responsible for these effects are not well addressed. Using methods of computational biology for target identification, we tested more than 200 PAs. We used a machine-learning approach that applies structural similarity for target identification, ChemMapper, and SwissTargetPrediction. The predicted target with high probability was muscarinic acetylcholine receptor M1. The predicted interactions between this target and PAs were further studied by molecular docking-based binding energies using AutoDock and VinaLC, which revealed good binding affinities. The PAs are bound to the same binding pocket as pirenzepine, a known M1 antagonist. These results were confirmed by in vitro assays showing that PAs increased the levels of intracellular calcium. We conclude that PAs are potential acetylcholine receptor M1 antagonists. This elucidates for the first time the serious neuro-oncological toxicities exerted by PA consumption.

## 1. Introduction

Agricultural plants serve as sources of numerous nutritional and medicinal products (e.g., honey, black tea, green tea, medicinal herbal teas, and herbal infusions). During cultivation and harvesting of crops, cross-contamination with pyrrolizidine alkaloid (PA)-containing weeds may occasionally occur [1,2]. Around 6000 plant species, constituting 2% of all flowering plants, are believed to possess the ability to produce PAs. Currently, PAs have been identified in over 600 distinct species, primarily hailing from the Asteraceae (Compositae), Boraginaceae, and Fabaceae (Leguminosae) families. 

The mechanism of action of pyrrolizidine alkaloid (PA) toxicity involves the intricate processes of toxicokinetics, biotransformation, and genotoxicity. The contamination of products with even low PA doses provokes hepatotoxicity and carcinogenesis [3,4,5].

**Figure 1:**
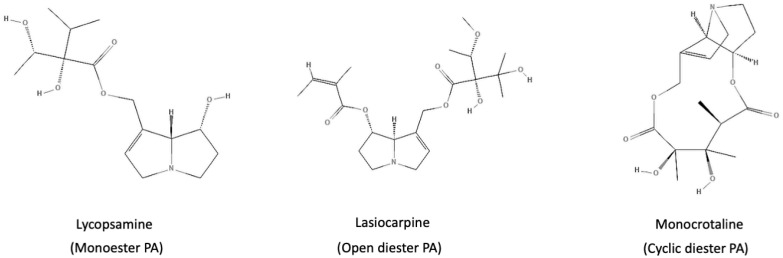
Examples of pyrrolizidine alkaloids with different kinds of esterification (monoester, dieter, and cyclic diester).

Pyrrolizidine alkaloids serve as a defense mechanism for plants against bacterial or fungal infections and animal or insect attacks. Within plants, for example, the Senecio spp., these alkaloids, synthesized in roots, are translocated to shoots and stored in preferred locations, forming a crucial aspect of plant–insect interactions [6,7]. The basic chemical structure of PAs consists of a necine base, often with one or more necine acids linked to the necine base via ester bonds [8]. PAs have been classified into four different categories according to their necine bases: retronecine-, heliotridine-, otonecine-, and platynecine-type PAs [9]. An alternative classification system is based on the number of ester bonds linked with the necine base and their overall structure (Figure 1). According to this system, four groups were established: mono-, di-, macrocyclic diesters, and “others”. The category “others” is a collection of PAs that do not fit into the three previously mentioned groups [10].

Several reports on the contamination of herbal products, including teas and herbal medicines with small traces of PAs, have raised public attention in newspapers and TV [11]. Some studies on tea products reported that up to 86% of the tested samples were contaminated with PA. In fact, most of these contaminated products are consumed as food, which means they are subject to fewer regulations. In addition, the contaminated herbs and herbal mixtures are commonly consumed by pregnant, lactating women and infants as herbal remedies to relieve symptoms of morning sickness and digestive problems or for their calming effect on the body. Involuntary PA consumption represents a serious health problem, especially if consumed by infants and pregnant or lactating women, because this category is more vulnerable to toxicity [12]. The Panel on Contaminants in the Food Chain of the European Food Safety Authority (EFSA CONTAM) proposes a set of 17 PAs to be monitored in food. It is recommended to monitor additional PAs to better understand the prevalence of PAs in food and to protect customers from PA intake [13].

A major complication of PAs is the development of liver cirrhosis and hepatocarcinogenesis as a result of long-term use of PA-containing plant products. The toxicity resulting from PAs underscores a lack of oversight in monitoring the safety of traditional medicine. Particular emphasis should be placed on people with underlying liver diseases or those concurrently using medications that induce specific cytochrome P450s, as they may be more susceptible to PA-induced hepatotoxicity [14]. It has been found that the disruption of crucial signaling pathways linked to DNA damage repair and cell cycle regulation in the transfected hepatocytes by the PAs, as revealed by the transcriptome profiling of altered gene expression, may account for the carcinogenic consequences of PAs. In addition, one unique mechanism of PAs is the disruption of chromosomal congression, which may also play a role in PA-mediated carcinogenesis [15]. PAs are also responsible for other serious health problems, including chronic diseases such as a variety of cancers, PAH, progressive liver disease leading to cirrhosis, congenital anomalies, and a combination of these could occasionally be brought on, started, or accelerated by existing sporadic, low levels of dietary exposure to PAs. Particularly distinctive and suggestive reactions to exposure to dehydroPA are PAH and HSOS. Any chronic illness case with evidence of one or both of these extremely uncommon co-occurring disorders may suggest dietary PAs as a potential cause [16]. PAs exert varying levels of neurotoxicity, which are also considered serious adverse effects of PA consumption [17].

The chemical diversity of PAs and the wide range of toxic reactions in the human body are clues that PAs exert broad-spectrum toxicity by multiple rather than single targets. The application of computational predictions based on similarity searches represents an approach to identifying unknown molecular interactions between small chemical molecules and proteins. In this study, we used target prediction programs to explore possible molecular targets of PAs. The application of computational predictions was based on similarity searches, where ligands with similar chemical structures and molecular shapes have a higher probability of sharing common protein targets [18]. We provided insight into the mechanism of action of PA by elucidating its binding to the muscarinic acetylcholine receptor M1, revealing an association with the neurotoxic effects of PA.

## 2. Results

### 2.1. Target Prediction Analysis 

**Figure 2:**
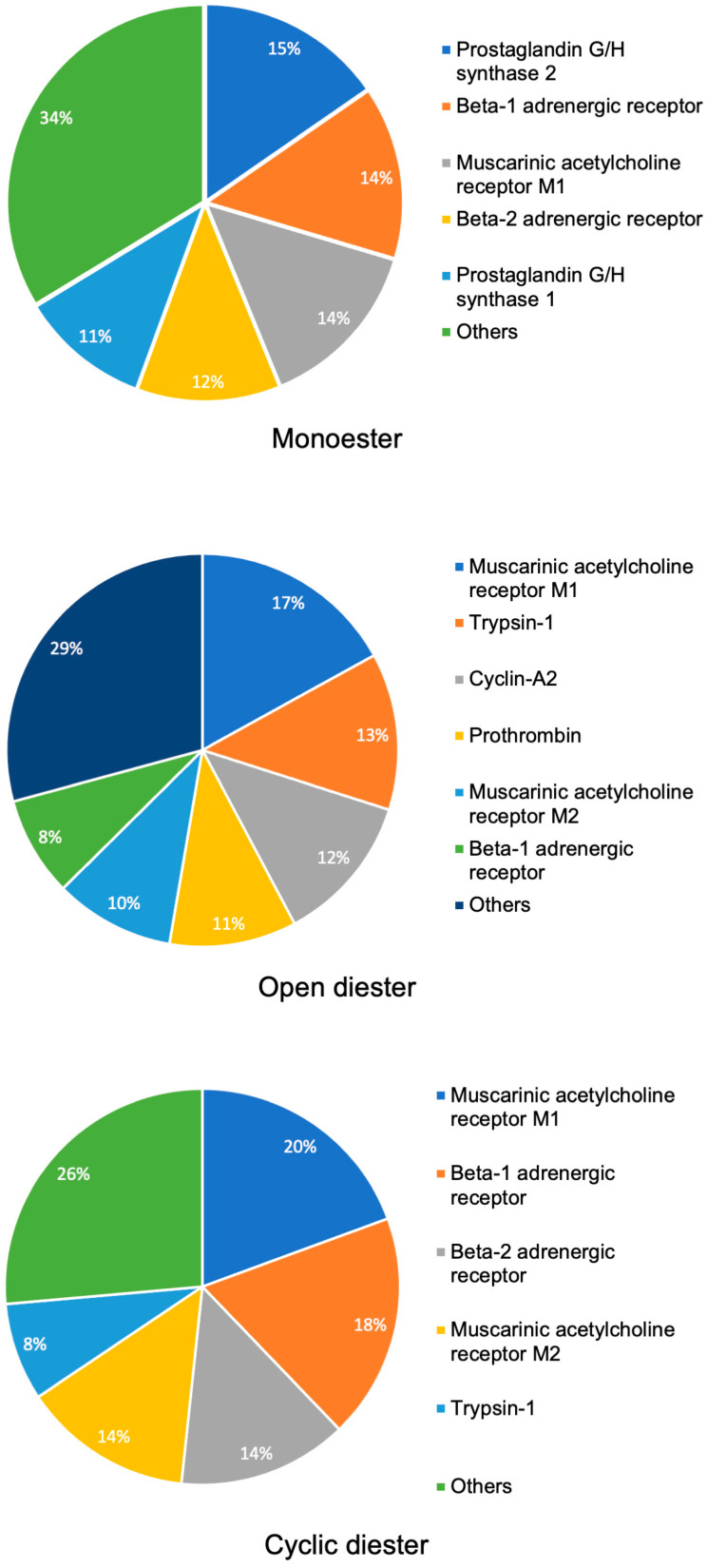
Most common targets for approximately 250 pyrrolizidine alkaloids based on the top target predictions using ChemMapper software. The incidence rate is depicted in percentages, and a 2% cutoff is included. The pie charts are divided into their respective categories: monoester, diester, cyclic, and others. ChemMapper: https://www.lilab-ecust.cn/chemmapper/help.html, accessed on 18 May 2021 [19].

In our target prediction analysis using ChemMapper [19], we identified potential binding targets for three groups of PAs. These predictions were obtained through a 3D similarity search. For the monoester group, prominent targets included prostaglandin G/H synthase 2, β-1 adrenergic receptor, muscarinic acetylcholine receptor M1, β-2 adrenergic receptor, and prostaglandin G/H synthase 1. Open diester PAs exhibited high probabilities of binding to targets such as muscarinic acetylcholine receptor M1, trypsin-1, cyclin-A2, prothrombin, muscarinic acetylcholine receptor M2, and β-1 adrenergic receptor. Cyclic diester PAs, on the other hand, showed potential interactions with muscarinic acetylcholine receptor M1, β-1 adrenergic receptor, β-2 adrenergic receptor, muscarinic acetylcholine receptor M2, and trypsin-1. The results are shown in Figure 2.

**Figure 3:**
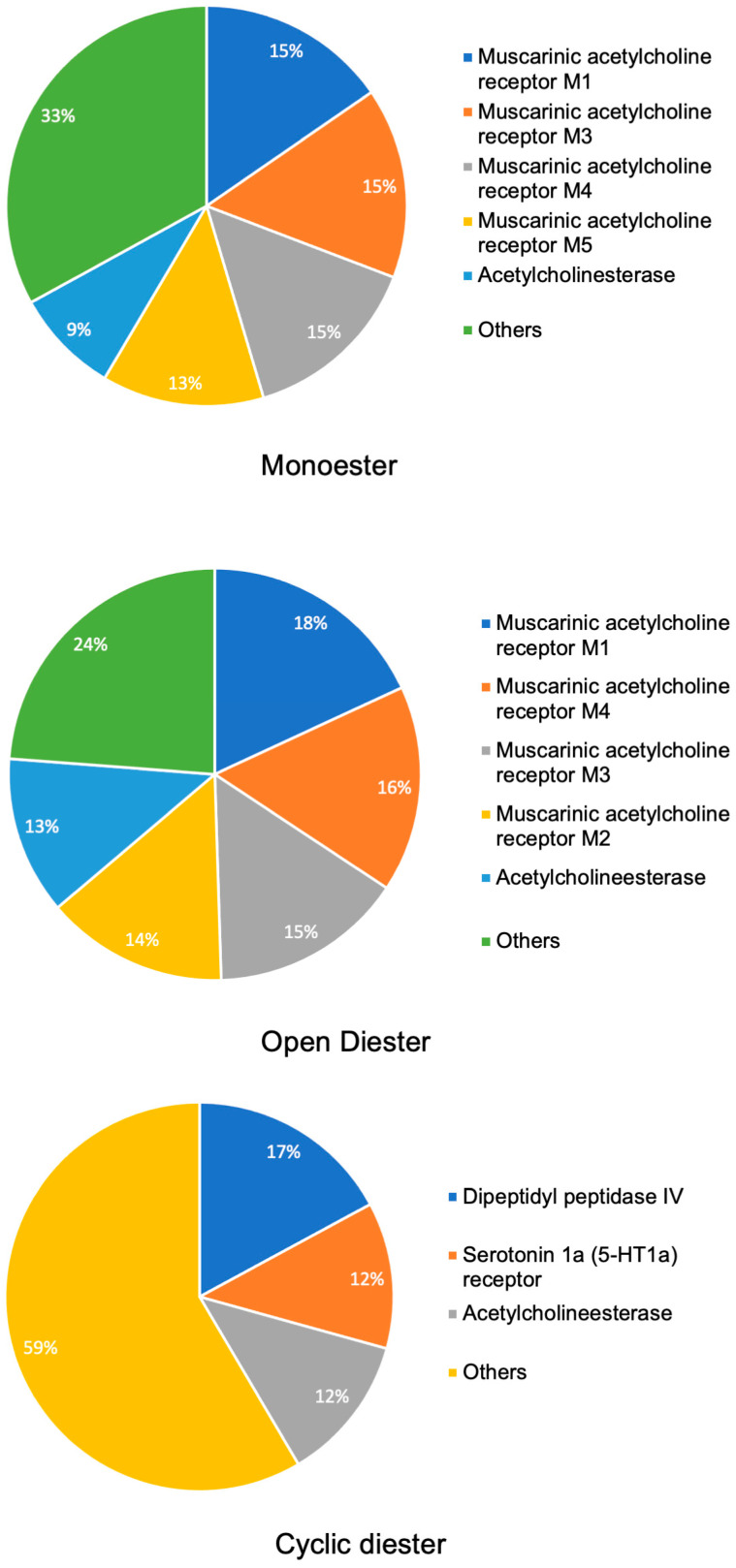
Most common targets for approximately 250 pyrrolizidine alkaloids based on the top target predictions using SwissTargetPrediction software. The incidence rate is depicted in percentages, and a 2% cutoff is included. SwissTargetPrediction: http://www.swisstargetprediction.ch [20].

Employing SwissTargetPrediction [20], the results shown in Figure 3 corroborated and extended our findings. The monoester group was predicted to target muscarinic acetylcholine receptors M1, M3, M4, M5, and acetylcholinesterase. Open diester PAs were implicated in interactions with muscarinic acetylcholine receptors M1, M4, M3, M2, and acetylcholinesterase. Lastly, cyclic diester PAs exhibited binding to dipeptidyl peptidase IV, serotonin 1a (5-HT1a) receptor, and acetylcholinesterase.

Across both ChemMapper and SwissTargetPrediction results, the muscarinic acetylcholine receptor M1 (CHRM1) emerges as the most frequently predicted target among all three PA groups, suggesting a robust and consistent association that merits particular attention in our further investigations.

### 2.2. Molecular Docking

The docking results of various pyrrolizidine alkaloids (PAs) with the CHRM1 receptor are summarized in Table 1. Notably, the comparison of results with pirenzepine, a recognized selective M1 antagonist used as a control compound, revealed insightful findings.

Pirenzepine exhibited the most robust binding energy (−7.42 kcal/mol), engaging with several amino acids in the orthosteric binding site, including Trp101, Leu102, Tyr106, Cys178, Leu183, and Trp400. In contrast, other PAs showed varying degrees of binding energies and distinct amino acid interactions. Senecionine exhibited the strongest binding energy at −7.36 kcal/mol, engaging with Trp101, Leu102, Tyr106, Cys178, Tyr179, Ile180, Leu183, Thr189, and Tyr404. Following closely, riddelliine displayed a binding energy of −7.17 kcal/mol, interacting with Ile180, Leu183, Ser184, Thr189, and Tyr404. Lasiocarpine, with a binding energy of −6.19 kcal/mol, demonstrated interactions with a diverse set of amino acids, including Tyr82, Trp101, Leu102, Tyr106, Tyr179, Ile180, Glu180, Leu183, Tyr381, Trp400, Glu401, and Tyr404.

Monocrotaline exhibited a binding energy of −6.96 kcal/mol, interacting with Tyr85, Leu102, Tyr106, Cys178, Tyr179, Ile180, and Leu183. Echimidine, with a binding energy of −6.04 kcal/mol, engaged with Tyr82, Tyr85, Tyr106, Cys178, Tyr179, Thr189, Ile180, Leu183, and Tyr404. Echiumine (−6.06 kcal/mol) displayed interactions with Tyr82, Tyr85, Tyr106, Cys178, Tyr179, Ile180, Leu183, Thr189, Trp400, Glu401, and Tyr404. Despite having a positive binding energy of 4.74 kcal/mol, otonecine exhibited interactions with Tyr82, Tyr85, Gin177, Cys178, Glu401, and Tyr404.

**Figure 4:**
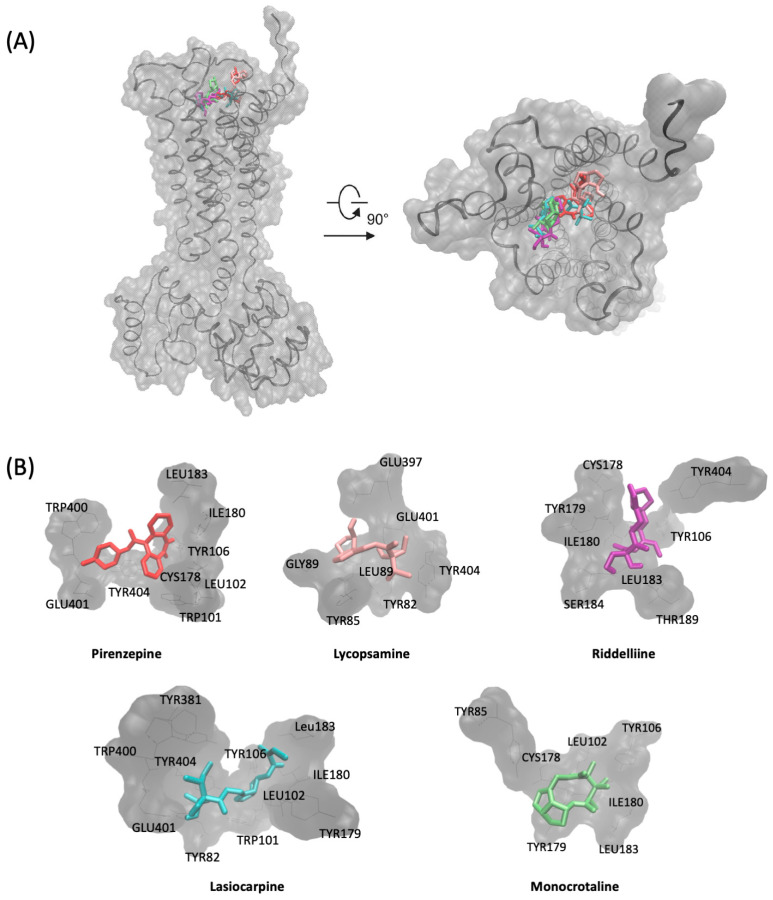
Molecular docking of selected pyrrolizidine alkaloids and pirenzepine to the muscarinic acetylcholine receptor M1. (**A**) The 3D structure of the muscarinic acetylcholine receptor M1 (grey) and the PAs lycopsamine (pink), lasiocarpine (blue), monocrotaline (green), riddelliine (purple), and the known antagonist pirenzepine (red). The compounds were visualized in their lowest energy conformation based on molecular docking using AutoDock. (**B**) Binding pockets of the ligands lycopsamine, lasiocarpine, monocrotaline, riddelliine, and pirenzepine. The binding pocket is visualized with the interacting amino acids based on molecular docking using AutoDock.

Overall, the results shown in Figure 4 suggest that PAs interact with CHRM1 through a combination of hydrophobic and hydrogen bonding interactions. While the specific amino acids involved varied among PAs, key residues such as Trp101, Leu102, Tyr106, Cys178, and Tyr179 were consistently implicated. The binding affinities of diester PAs (echimidine, lasiocarpine, and echiumine) and cyclic PAs (riddelliine, monocrotaline, and senecionine) were noteworthy, indicating potential agonistic or antagonistic effects on CHRM1. The shared binding pocket with pirenzepine further suggests the relevance of these PAs in modulating CHRM1 activity.

### 2.3. Determination of Agonistic or Antagonistic Functions by a Calcium Efflux Assay

**Figure 5:**
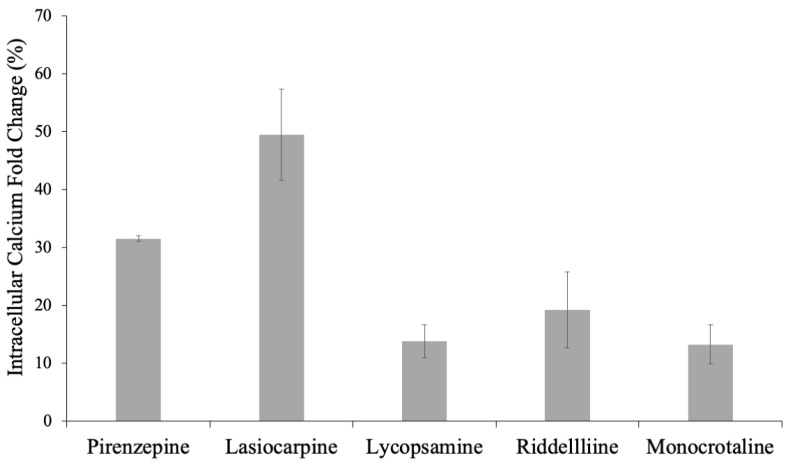
Fold change in intracellular calcium of CHO-K1 cells treated with lycopsamine, lasiocarpine, monocrotaline, riddelliine, and pirenzepine.

The assessment of calcium accumulation in CHO-K1 cells following treatment with various compounds revealed intriguing findings. Lasiocarpine, a pyrrolizidine alkaloid (PA), induced a substantial 49.5% (±7.8) fold increase in calcium levels, surpassing the effect observed with pirenzepine, the positive control, which showed a 31.5% (±0.5) fold increase. This outcome underscores the potency of lasiocarpine in elevating cellular calcium concentrations. Notably, lycopsamine, riddelliine, and monocrotaline exhibited moderate activities, with fold changes of 13.7% (±2.8), 19.2% (±6.5), and 13.2% (±3.4), respectively (Figure 5).

If the CHRM1 receptor is activated, PAs link up with Gq/11 proteins. This triggers an enzyme called phospholipase C, which unlocks calcium storage within the cell, causing calcium release into the cytoplasm [21]. Therefore, we suggest that PAs act in a CHRM1 antagonistic manner as a result of the increase in calcium accumulation.

## 3. Discussion

In silico toxicology is an emerging new field facilitating the prediction and understanding of off-target effects of drugs or toxins [22]. It is paramount to chemical, pharmaceutical, and nutritional industries, as well as regulatory agencies, to maintain high customer safety levels and to develop guidelines for risk minimization. Also, mastering this field potentially decreases the demand for animal experiments. Combining methods indispensable for toxicity prediction, e.g., quantitative structure–activity relationship calculations, structural alerts, read-across, and deep learning, is a must. Information can be obtained as mutagenicity, toxicological pathways, organ toxicity, and various side effects [23,24].

In this investigation, we applied in silico toxicology to determine the unknown mechanisms mediating the toxic effects of PAs. We used machine learning-based prediction webservers that apply either 2D or 3D quantitative structure–activity relationships to define possible targets of PAs. Based on the obtained results, we proceeded to investigate the potential target in more detail: the muscarinic acetylcholine receptor M1. Of course, the selection for these targets was based on the analysis of probability across all classes of PAs. Yet, our motivation was raised due to a possible connection between these targets and PA neurotoxicity. Toxicity of the nervous system as a result of PA poisoning was reported by WHO-IPCS (1988) [25].

Several studies have documented neurological disorders in animals associated with pyrrolizidine alkaloid (PA) intake. On Easter Island, a discernible correlation emerged between the development of fetal neurological signs, anorexia, and weight loss in a group of horses consuming PA-containing plants, specifically Crotalaria grahamiana. These behaviors, locally labeled as “Crazy Horse” (CH), were accompanied by hepatotoxic effects attributed to elevated PA intake. The research conducted from 2010 to 2015 further investigated this link, revealing a strong association between the consumption of C. grahamiana, particularly its monocrotaline content, and hepatic encephalopathy in affected horses. The evidence suggests a causal relationship between pyrrolizidine alkaloid ingestion and the manifestation of CH disease on Easter Island [26]. Also, CNS toxicity in animals is linked to monocrotaline poisoning from Crotalaria plants. Metabolic interactions between neurons and astrocytes are vital for brain development and detoxification. Despite traditional medicinal use, Crotalaria is recognized as toxic. Studies connect CNS toxicity to monocrotaline, a pyrrolizidine alkaloid causing damage, with hepatic and pulmonary effects mediated by P450 systems [27].

Different PAs exert variable levels of neurotoxicity depending on different factors such as chemical structure, lipophilicity, metabolism rate, and blood–brain barrier permeability [17]. Yet, the exact mechanism of nervous system toxicity has not been fully elucidated.

Our molecular docking protocol has shown high precision and significant correlation to in vitro experiments. This was previously documented in a number of articles in the field of drug discovery and toxicology [28,29]. We applied the same workflow again to verify the binding of PAs to the predicted targets. The results showed that the selected examples from the chemical classes have a very high affinity to bind to muscarinic acetylcholine receptor M1 compared to a known control drug.

We suggest that the interaction between different classes of PAs and CHRM1 is a major cause of the above-mentioned neurotoxicity and complications of PAs.

The cholinergic receptor muscarinic 1 (CHRM1) belongs to a large family of protein-coupled receptors (GPCRs). This receptor is the most expressed in the central nervous system, with a high concentration in the cortical region; the location of this receptor includes the amygdala, hippocampus, and basal ganglia. This receptor is activated with the binding of the neurotransmitter acetylcholine. This receptor is involved in many brain functions, including motor control, learning, attention, cognition, memory, and controlling of sleep and wake-up cycle [30]. The disruption of the activity of the acetylcholine receptors has been associated with diseases of the nervous system, such as Parkinson’s disease and Alzheimer’s disease [31]. Overactivation of the muscarinic receptor at the CNS and neuromuscular junction as a result of exposure to nerve agents can lead to severe symptoms due to excessive accumulation of acetylcholine; the symptoms include abnormal behavior, confusion, slurred speech, mental status, weakness, and paralysis [32,33].

The in vitro analysis using a calcium efflux assay in CHO-K1 cells stably transfected with the CHRM1 validated our in silico suggestions. Four tested PAs showed M1 antagonistic effect, especially lasiocarpine. The literature extensively outlines the toxic effects of lasiocarpine and riddelliine, emphasizing genotoxic and hepatotoxic impacts. Using in vitro genotoxicity data and PBK modeling, one study predicts in vivo genotoxicity in rats, effectively assessing PAs lacking sufficient in vivo data through histone protein H2AX phosphorylation [34]. Yet, as highlighted here, more studies are required to test their neurologic effect.

Regulatory authorities consider PAs as major contaminants that should be regulated in herbal products. Their concern is to keep their presence below the minimum limits that will not be harmful for human consumption. The Federal Institute for Risk Assessment (BfR) regularly assesses food contamination by 1,2-unsaturated pyrrolizidine alkaloids, which can have genotoxic and carcinogenic effects. Using the margin of exposure (MOE) concept, recent data from 2015 to 2019 reveals a significant reduction in PA levels, especially in teas. However, caution is advised, as certain food groups like herbs/spices may contribute to exposure, necessitating ongoing efforts to minimize PA levels across all food groups [35]. In a previous study, we established the dose-dependent genotoxic effects of PAs, emphasizing transcriptomic analysis as a key technique for setting limit values. We applied this approach to assess low concentrations of PAs in human liver cells, revealing disruptions in cell cycle regulation and DNA damage repair pathways. The study highlights the significance of transcriptomic, cell cycle, and immunofluorescence analyses in comprehending the biological effects of PA exposure on liver cells [15].

Understanding the broad-spectrum toxic effects of PAs is crucial for implementing limit values in practical use to prioritize the toxic effects of different PAs and to determine limit concentrations of each effect.

## 4. Materials and Methods

### 4.1. Collection of Chemical Structures and Classification of PAs

To identify various PAs, we conducted a literature search on PubMed, employing the keywords "pyrrolizidine alkaloids" and filtering the results to include review articles. A total of 250 different PAs with available structural information were identified. Subsequently, their chemical structures were classified based on their number of ester bonds linked with a necine base and their overall structure. Four groups were established: mono-, di-, macrocyclic diesters, and others. The PAs’ three-dimensional (3D) structures were downloaded as SDF files from PubChem.

### 4.2. Target Prediction with ChemMapper

ChemMapper is a web server used for target prediction through a ligand similarity search (https://www.lilab-ecust.cn/chemmapper/help.html). Biological function, protein targets, and pharmacology actions of 300,000 drug-like molecules were collected from public databases (ChEMBL, DrugBank, BindingDB). The program uses a 3D similarity calculation method called SHAFTS, which combines the strength of molecular shape superposition and chemical feature matching. This method is more accurate compared to regular 2D fingerprint-based similarity and other 3D similarity methods [19]. Scores range from 0 to 1 to rank the predicted targets. Each chemical structure of the 250 PAs was uploaded to the web server, and the top 5 targets were predicted.

### 4.3. Target Prediction Using SwissTargetPrediction

SwissTargetPrediction is a web server that identifies novel targets for small chemical molecules (http://www.swisstargetprediction.ch). It applies both 2D and 3D similarity measures with known ligands. Results are represented in scores ranging between 0 and 1 to rank the target prediction probability [20]. Each of the 250 obtained structures of PAs was subjected to the web server, and the top 5 targets were predicted.

### 4.4. Analysis of Target Prediction

The output from the target prediction servers ChemMapper and SwissTargetPrediction were analyzed using pandas (version: 1.2.3). The target counts were extracted for each platform and each chemical subgroup of PAs. The results were visualized with a cut-off value of 2%.

### 4.5. Molecular Docking

We applied molecular docking using AutoDock 4.2. and VinaLC (v1.3.0) to verify the binding between PAs and the predicted targets [36]. The 3D ligand structures were downloaded from PubChem (National Center of Biotechnology Information, MD, USA) as standard data files. Open Babel was used to transform all the ligands from SDF files to PDBQT files. The crystal structure of muscarinic acetylcholine receptor 1 (MAChRs) (PDB code: 6WJC) was downloaded from the Protein Data Bank (http://www.rcsb.org/) as a PDB file. AutoDockTools 1.5.7 was used to prepare the molecular docking process. All PDB files were converted to Protein Data Bank Partial Charge and Atom Type (PDBQT) files.

A grid box was set to include the all-binding pocket for each protein. MAChR was executed with the grid box at x = 20.338, y = 19.360, and z = 4.042 with the number of grid points (npts) of 90 in x, 90 in y, and 90 in z. For nAChR, the center of the grid box was set at x = 4.226, y = 1.361, and z = −0.889 with npts of 100 in x, 80 in y, and 90 in z.

Molecular docking and estimation of binding affinities were carried out using an HPC “snakemake workflow” that automated structural-based screening stages (https://github.com/snakemake-workflows/structure-based-screening). The workflow employs Vina LC (version 1.3.0) for docking, Open Babel (version 3.0.0) for ligand energy minimization, and Biopython (version 1.75). Also, the AutoDock built-in Lamarckian Algorithm has been used for the calculation, and the interaction between amino acids and proteins was identified with AutoDockTools. Visual Molecular Dynamics 1.9.3 (VMD) was used to create the visualizations (http://www.ks.uiuc.edu/Research/vmd/).

Parts of this analysis have been conducted using the supercomputer MOGON II and advisory services offered by the Johannes Gutenberg University Mainz (http://www.hpc.uni-mainz.de), which is a member of the AHRP (Alliance for High-Performance Computing in Rhineland Palatinate, http://www.ahrp.info) and the Gauss Alliance e.V.

### 4.6. Determination of Agonistic/Antagonistic Activity by Calcium Efflux Assay

CHO-K1 cells stably transfected with the CHRM1 were purchased from the cDNA Resource Center (Bloomsburg University of Pennsylvania, Bloomsburg, PA, USA). Cells were grown in Ham’s F-12 Nutrient Mix, GlutaMAX™ Supplement medium (Thermo Fischer Scientific, Waltham, MA, USA) supplemented with heat-inactivated 10% fetal bovine serum (FBS), 100 U/mL penicillin, and 100 μg/mL streptomycin (Invitrogen, Darmstadt, Germany). Cells were treated with 250 µg/mL geneticin to maintain the transfected phenotype.

Levels of intracellular calcium were assayed using the FluoForte calcium assay (ENZ-51017; Enzo Life Sciences, Lörrach, Germany) according to the manufacturer’s protocol. CHO M1 cells (500,000 cells/mL) were seeded into 6-well plates and incubated overnight. Afterward, cells were treated for 1 h with 20 µM of four PAs (lycopsamine, lasiocarpine, monocrotaline, and riddellliine) and pirenzepine (20 and 40 µM) was used as a positive control. Then, FluoForte dye was added for 45 min at 37 °C. Cells were then incubated for 15 min at room temperature before fluorescence was measured on a BD Accuri™ C6 flow cytometer (Becton-Dickinson, Heidelberg, Germany).

## 5. Conclusions

This study employed in silico toxicology approaches, including machine learning-based prediction webservers and molecular docking, to unravel the potential molecular targets and toxic effects of PAs. The findings underscored the neurotoxic potential of PAs, particularly through their high affinity binding to CHRM1. The calcium efflux assay further validated these in silico predictions, revealing M1 antagonistic effects. The results shed light on the broad-spectrum toxicity of PAs and their potential impact on the nervous system. The study emphasizes the importance of understanding the toxic effects of PAs for regulatory considerations, suggesting the need for limit values in herbal products to safeguard against the diverse health risks associated with PA consumption. Additionally, the research underscores the significance of further studies to explore the neurologic effects of specific PAs, contributing to a comprehensive understanding of their overall toxicity profile.

## Figures and Tables

**Table 1:** Molecular docking of PAs to muscarinic acetylcholine receptor 1 (MAChRs) (PDB: 6WJC).

Compound	Mean Binding Energy–VinaLC (kcal/mol)	pKi/nM	Amino Acids Involved in Interactions	Amino Acids Involved in H-Bonds
Pirenzepine	−7.42 (±0.32)	2.74 × 10^3^ (±0.09 × 10^3^)	Trp101, Leu102, Tyr106, Cys178, Leu183, Trp400, Glu401, Tyr404	
Heliotrine	−5.31 (±0.34)	36.27 × 10^3^ (±7.08 × 10^3^)	Tyr82, Tyr85, Cys98, Trp101, Tyr106, Cys178, Tyr179, Ile180, Leu183, Tyr381, Tyr404	Tyr106, Cys178, Ile180
Rinderine	−5.62 (±0.12)	39.69 × 10^3^ (±7.08 × 10^3^)	Leu102, Tyr106, Cys178, Tyr179, Ile180, Leu183, Thr189, Tyr381, Tyr404	Ile180 (2), Tyr381
Lycopsamine	−5.71 (±0.13)	4.44 × 10^6^) (±0.68 × 10^6^)	Tyr82, Tyr85, Leu86, Gly89, Glu397, Glu401, Tyr404	Tyr82, Glu397, Glu401
Echimidine	−6.04 (±0.29)	21.61 × 10^3^ (±8.21 × 10^3^)	Tyr82, Tyr85, Tyr106, Cys178, Tyr179, Ile180, Leu183, Thr189, Trp400, Glu401, Tyr404	Thr189, Glu401
Lasiocarpine	−6.19 (±0.23)	8.91 × 10^3^ (±1.53 × 10^3^)	Tyr82, Trp101, Leu102, Tyr106, Tyr179, Ile180, Leu183, Tyr381, Trp400, Glu401, Tyr404	Ile180, Glu401
Echiumine	−6.06 (±0.19)	16.99 × 10^3^ (±2.51 × 10^3^)	Tyr82, Tyr85, Tyr106, Cys178, Tyr179, Ile180, Leu183, Thr189, Trp400, Glu401, Tyr404	Tyr106
Riddelliine	−7.17 (±0.42)	4.67 × 10^3^ (±0.04 × 10^3^)	Tyr106, Cys178, Tyr179, Ile180, Leu183, Ser184, Thr189, Tyr404	Ile180, Leu183
Senecionine	−7.36 (±0.42)	8.44 × 10^3^ (±0.0)	Trp101, Leu102, Tyr106, Cys178, Tyr179, Ile180, Leu183, Thr189, Tyr404	Tyr404
Monocrotaline	−6.96 (±0.3)	1.55 × 10^6^ (±0.0)	Tyr85, Leu102, Tyr106, Cys178, Tyr179, Ile180, Leu183	Tyr106
Casuarine	−5.06 (±0.18)	9.84 × 10^6^ (±0.66 × 10^6^)	Tyr85, Leu86, Gly89, Gln177, Glu397, Glu401	Leu86, Gln177, Glu397(2), Glu401
Tussilagine	−5.16 (±0.7)	3.67 × 10^6^ (±0.04 × 10^6^)	Tyr106, Tyr179, Ile180, Leu183, Thr189	Ile180
Crotanecine	−4.57 (±0.15)	13.20 × 10^6^ (±0.26 × 10^6^)	Tyr85, Leu86, Gly89, Glu397, Glu401	Glu397(2), Glu401
Otonecine	−4.74 (±0.16)	10.20 × 10^6^ (±0.06 × 10^6^)	Tyr82, Tyr85, Gln177, Cys178, Glu501, Tyr404	Tyr85, Gln177, Glu501

## Data Availability

The datasets used and/or analyzed during the current study are available in IRODs (https://irodsweb.zdv.unimainz.de/irodsrest/rest/fileContents/zdv/project/m2_jgusmitt/antagonistic_action_muscarinic_acetylcholine_receptor_M1.zip?ticket=I8jMe9xpUYI8fC8).
